# Successful Modified Therapy in a Patient with Probable Infection-Associated Hemophagocytic Lymphohistiocytosis

**DOI:** 10.1155/2019/9781065

**Published:** 2019-09-08

**Authors:** Carl L. Kay, Matthew J. Rendo, Paul Gonzales, Sead G. Beganovic, Magdalena Czader

**Affiliations:** ^1^Brooke Army Medical Center, Ft. Sam Houston, TX, USA; ^2^Hematology-Oncology, Indiana University School of Medicine, Indianapolis, IN, USA; ^3^Hematopathology, Indiana University School of Medicine, Indianapolis, IN, USA

## Abstract

Hemophagocytic lymphohistiocytosis (HLH) is a rare, hyperinflammatory syndrome characterized by clinical signs and symptoms of extreme inflammation. In adults, HLH is typically a complication of infections, autoimmune diseases, and malignancies. While the disease is often fatal, classic management of HLH revolves around early diagnosis and initiation of protocolized therapy. We present a case of a previously healthy 56-year-old female who developed distributive shock requiring intubation, vasopressors, and continuous venovenous hemofiltration. In the setting of multiple infectious syndromes, severe cytopenias, and rising direct hyperbilirubinemia, her diagnosis of HLH was confirmed. Therapy was initiated with dexamethasone and two doses of reduced-intensity etoposide based on the patient's clinical course. Over the next few weeks, she continued to improve on dexamethasone monotherapy and has maintained remission up to the present with complete resolution of her cytopenias and return of baseline renal function. Our case highlights the variability in the management of probable infection-associated HLH (IHLH) with a good patient outcome. We demonstrate the potential to treat IHLH with partial protocols and minimal chemotherapeutics.

## 1. Introduction

Hemophagocytic lymphohistiocytosis (HLH) is a rare, hyperinflammatory syndrome characterized by clinical signs and symptoms of extreme inflammation [1]. There are revised criteria for establishing the diagnosis of HLH from the Histiocyte Society based upon the clinical, laboratory, and histopathologic findings (Table 1). HLH is not a single disease but instead represents a collection of disease processes with a similar end-stage phenotype [2]. HLH has been described as a primary disorder (i.e., familial or genetic HLH) and also secondary to other disease processes. While both forms of HLH are associated with high morbidity and mortality, secondary HLH occurs in the setting of infectious, malignant, rheumatologic, or metabolic conditions [3]. Occasionally, an inciting trigger is not identified and HLH is considered idiopathic [4].

**Figure 1:**
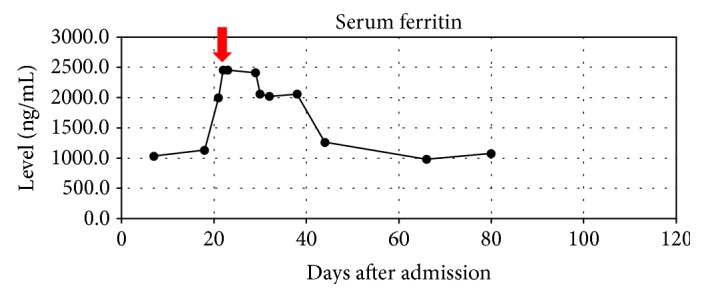
Overview of patient ferritin levels with indication of dexamethasone and etoposide initiation.

**Figure 2:**
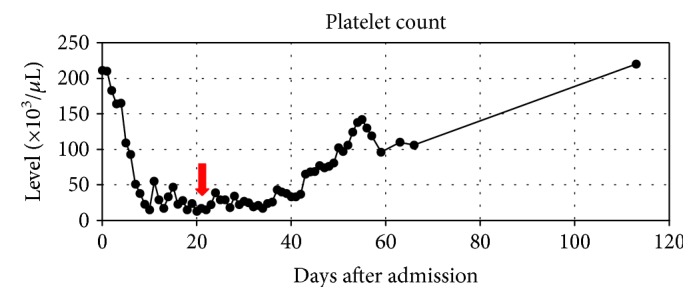
Overview of patient platelet count with indication of dexamethasone and etoposide initiation.

**Figure 3:**
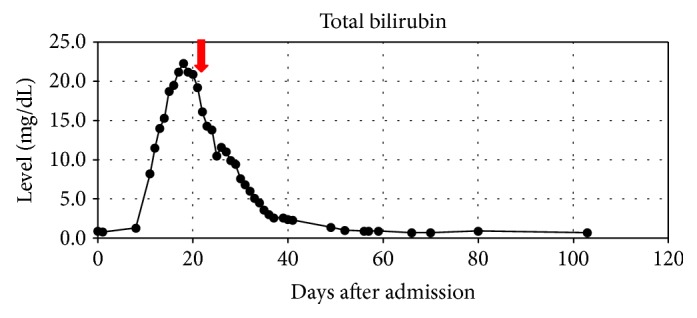
Overview of patient total bilirubin levels with indication of dexamethasone and etoposide initiation.

**Figure 4:**
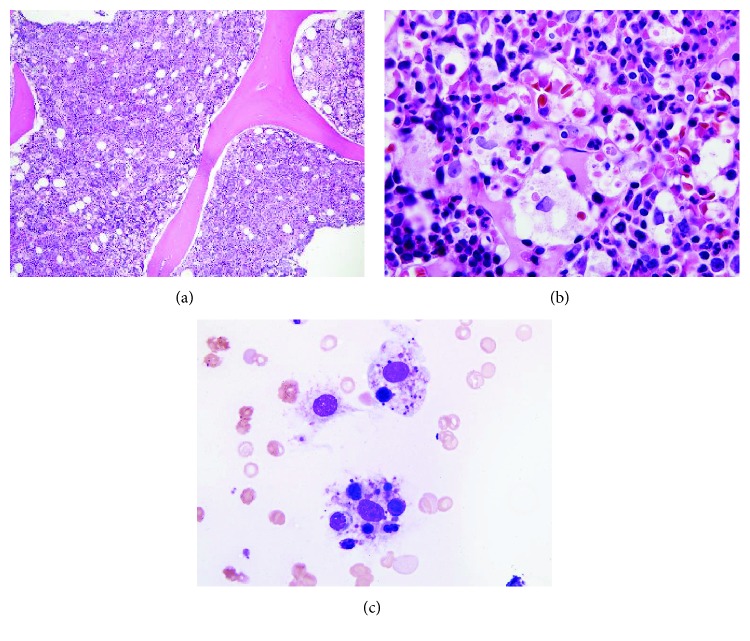
(a) Markedly hypercellular bone marrow with left-shifted granulopoiesis, increased erythropoiesis with prominent erythroid islands, decreased megakaryopoiesis, and prominent hemophagocytosis (bone marrow biopsy, H&E, 200x). (b) Clusters of histiocytes with hemophagocytosis (bone marrow biopsy, H&E, 400x). (c) Bone marrow aspirate was hemodilute; however, it showed focal hemophagocytosis (bone marrow aspirate smear, Wright-Giemsa stain, 1000x).

We will present a case of septic shock with subsequently diagnosed HLH treated successfully without conventional therapy. We provide an overview of the clinical presentation, hospital timeline, and antimicrobial therapy in Tables 2–5 and Figures 1–4.

## 2. Case Presentation

A 56-year-old African American female with no significant past medical history presented with one week of fatigue, nausea, vomiting, diarrhea, intermittent abdominal pain, and subjective fever. Laboratory investigation revealed a serum creatinine of 2.7 mg/dL with baseline creatinine of 0.8 mg/dL. Renal ultrasound was normal without evidence of obstruction. Computed tomography of the abdomen and pelvis was negative for acute intraabdominal pathology. She was admitted for supportive care and volume repletion for acute renal insufficiency.

Three days after admission, her gastrointestinal symptoms abated, but she developed a productive cough and leukocytosis. Chest X-ray showed increased interstitial markings consistent with atypical pneumonia versus interstitial edema, and she was started on antibiotics. She continued to worsen clinically developing fevers, tachypnea, hypotension, and atrial fibrillation with rapid ventricular response.

She had negative thromboembolic and viral infectious workups including HIV, Epstein-Barr virus, cytomegalovirus, parvovirus B19, human herpes virus-6, and endemic fungal infections. She met criteria for distributive shock, and antibiotic coverage was broadened. Platelet count dropped from 162 × 10^3^/*μ*L to 51 × 10^3^/*μ*L in the setting of progressive respiratory distress requiring intubation, vasopressor support, and continuous venovenous hemofiltration (CVVH).

Thrombocytopenic workup revealed elevated D-dimer, elevated haptoglobin, negative serotonin release assay, no red blood cell fragmentation, and only mildly reduced ADAMTS13 activity. Total bilirubin was 22.3 mg/dL with direct bilirubin of 18.4 mg/dL. Reimaging of the abdomen and pelvis revealed findings suggestive of acalculus cholecystitis, splenomegaly, and thickened gallbladder wall without obstruction. Hepatobiliary iminodiacetic acid (HIDA) scan was nondiagnostic. Endoscopic retrograde cholangiopancreatography (ERCP) was deferred due to severe thrombocytopenia. A transjugular liver biopsy revealed acute cholangitis, and she received percutaneous cholecystostomy and a course of meropenem.

With ongoing and nondiagnosed cytopenias, bone marrow biopsies were performed. First attempt bone marrow specimens were inadequate for analysis. Eventually specimens revealed macrophages with evidence of hemophagocytosis concerning for HLH. The patient met 7 of 8 criteria for establishing a diagnosis of HLH (fever, splenomegaly, cytopenias of at least two cell lines, hypertriglyceridemia, histiocytic hemophagocytosis, hyperferritinemia, and elevated soluble CD-25).

She was initiated on HLH-94 protocol with dexamethasone and etoposide. Given her significant renal impairment, etoposide was administered twice in the first week at 75% of the recommended dose (i.e., 40 mg/m^2^) based on a creatinine clearance less than 10 milliliters per minute. At the time of her second week of etoposide administration, the patient decompensated in the setting of an abdominal wall abscess associated with the aforementioned cholecystostomy drain. Continued etoposide protocol was deferred given the patient's immune suppression, dramatically worsened clinical status, and risk for toxicity in the setting of concomitant renal and liver dysfunction. Over the course of the next few weeks, she continued to improve without bleeding complications on dexamethasone monotherapy and supportive platelet and packed red blood cell transfusions.

Dexamethasone therapy was administered intravenously for the five weeks the patient remained hospitalized, then the patient was transitioned to oral dexamethasone for the remaining three weeks of the eight-week taper. She followed up monthly in the hematology/oncology clinic and maintained remission up to the present (14 months after admission) with complete resolution of cytopenias and return of baseline renal function.

## 3. Discussion

Repeatedly, the literature suggests that it is necessary to identify and diagnose HLH early to improve mortality. As a result of attempting to diagnose HLH early, diagnostic criteria have been criticized for their nonspecificity [5]. HLH is believed to be overdiagnosed due to these nonspecific diagnostic criteria [2]. Studies show that 60% of patients with severe sepsis and thrombocytopenia will demonstrate histiocytic hemophagocytosis [6]. Because these diagnostic features are not uncommon in severe sepsis, this suggests that there is much ambiguity about both the diagnosis and, subsequently, the management.

Given our patient's instability and multiple infectious syndromes, genetic testing for primary HLH was foregone. However, primary HLH was thought to be less likely due to the patient's age, no known familial mutations, and no family history of death of a young family member with unexplained fever [7]. Notably, primary HLH cannot be excluded especially considering literature suggesting that primary HLH is often set off by an infectious, malignant, or rheumatologic trigger [4, 8]. In the absence of known malignancy or rheumatologic disease, her multiple infectious syndromes were the most likely trigger of her HLH diagnosis.

Core to the uncertainty of infection-associated HLH (IHLH) management, it has previously been postulated that IHLH does not represent a distinct disease at all, but simply lies on the extreme end of the inflammatory spectrum of sepsis [9]. Benign histiocytic proliferation in the setting of infection was first described in 1979 by Risdall et al. [10]. Risdall et al. and other more recent literature have posited that patients do better with supportive care than the more radical and necessary treatment used in familial HLH [10, 11]. In contradiction to other literature suggesting that patients with IHLH should be treated on an HLH protocol [6, 12], we demonstrate the potential to treat IHLH with tailored therapy based on the patient's clinical scenario which may not require full-protocol chemotherapeutic agents for full recovery.

HLH guidelines for diagnosis and therapy are largely based on pediatric patient populations who demonstrated significant mortality benefit while following the therapeutic protocol [5, 13, 14]. Furthermore, the benefits of etoposide have been best described in viral IHLH [10–12, 15]. However, the literature continues to demonstrate the variability within the disease process, and there have been efforts to refine diagnosis and treatment based upon disease severity [3, 4]. It would seem that HLH subsets (i.e., adults and nonviral infection-associated HLH) may require therapeutic regimens different from those described in the HLH-94 and HLH-2004 protocols.

Our patient highlights the difficulties of managing such an adult patient with probable nonviral IHLH. There were several factors that made our case particularly challenging: multiple infectious syndromes, acute renal failure increasing the toxicity of etoposide, and delay in diagnosis due to inadequate first bone marrow sampling. Furthermore, our patient was at a very high risk of additional infections and further inflammation as she was immunosuppressed with chemotherapeutic agents per the HLH protocol. It is not clear that two doses of reduced-intensity etoposide caused clinical deterioration; however, full-protocol etoposide was not necessary for successful treatment. Notably, the continued dexamethasone monotherapy did not result in further deterioration.

While clinical courses and management of HLH vary widely, there are even case reports of HLH patients who improve spontaneously [16, 17]. Some patients diagnosed with secondary HLH survive with plasma exchange or immune globulin and without chemotherapy [18]. Given the paucity of literature regarding reduced-intensity, monotherapy, or alternative HLH treatment of nonviral IHLH, it is difficult to generalize suggested treatment regimens. However, our case suggests that a modified etoposide regimen may be appropriate in cases when there is clinical deterioration associated with etoposide initiation. Furthermore, dexamethasone monotherapy may be sufficient therapy for successful management in nonviral IHLH. This notion is somewhat contradictory to older observational studies indicating that rapid initiation of etoposide therapy is the only factor significantly associated with increased survival in selected adult patients with secondary HLH [11, 15]. Additionally, not all patients with secondary HLH require prolonged therapy, and, in many cases, treatment can be discontinued once their condition improves and the underlying condition has been treated [2]. The difficulty is identifying when chemotherapeutics are unnecessary and potentially detrimental. Additional reports of modified therapies for nonviral IHLH are needed to provide further guidance and protocols.

Our case highlights the variability in the management of adult IHLH with a good patient outcome. We demonstrate the potential to treat probable IHLH with a modified chemotherapeutic regimen. Given the infectious nature of our case, it would support the notion that IHLH does not represent a distinct disease at all, but IHLH may simply lie on the extreme end of the inflammatory spectrum of sepsis. Further study is needed to protocolize initiation of chemotherapeutics and deescalation of HLH-specific therapy especially in patients at high risk of infections.

## Figures and Tables

**Table 1:** Diagnostic criteria for HLH^∗^.

Fever ≥ 38.5°C
Splenomegaly
Peripheral blood cytopenia, with at least two of the following:
Hemoglobin < 9 g/dL (hemoglobin < 10 g/dL for infants < 4 weeks)
Thrombocyte count < 100 × 10^3^/*μ*L
Absolute neutrophil count (ANC) < 1000/*μ*L
Fasting triglycerides > 265 mg/dL and/or fibrinogen < 150 mg/dL
Hemophagocytosis in bone marrow, spleen, lymph node, or liver
Low or absent NK cell activity
Ferritin > 500 ng/mL
Elevated soluble CD-25 (soluble IL-2 receptor alpha) two standard deviations above age-adjusted laboratory-specific norms (or >2400 U/mL often cited in the literature)

^∗^≥5/8 criteria must be met for the diagnosis. Adapted from Jordan et al. [3]

**Table 2:** HLH diagnostic criteria and patient's presentation.

HLH-2004 diagnostic criteria	
Fever	+
Splenomegaly	+
Cytopenia of ≥2 cell lines	+
Triglyceride ≥ 254 mg/dL	+
Fribrinogen ≤ 150 mg/dL	−
Histiocytic hemophagocytosis	+
NK cell activity	Normal
Ferritin > 500 ng/mL	+ (2,455 ng/mL)
Elevated soluble CD-25	+ (2,661 U/mL)

**Table 3:** Overview of patient with HLH associated with severe sepsis and septic shock.

Features consistent with severe sepsis and septic shock	
Systemic inflammatory response syndrome	+
Infection	Unidentifiable
Hypotension requiring vasopressors	+
Acute respiratory distress syndrome requiring mechanical ventilation	−
Acute renal failure requiring dialysis	+
Altered mental status	+
Lactate > 2 mmol/L	+ (4.1 mmol/L)
Platelet count < 100 × 10^3^/*μ*L	+ (15 × 10^3^/*μ*L)
Disseminated intravascular coagulation	−
*Other clinical and laboratory features*	
Anasarca	−
Hepatomegaly	−
Peak AST (IU/L)	174
Peak ALT (IU/L)	192
Peak LDH (IU/L)	285
Peak total bilirubin (mg/dL)	22.3
Peak direct bilirubin (mg/dL)	18.4
Peak prothrombin time (s)	26.6
Nadir hemoglobin (g/dL)	5.7 (on hospital day 20)
Nadir absolute neutrophil count (cells/mm^3^)	1,600 (on hospital day 26)
Other features	Pericardial effusion, pneumonia, acalculus cholecystitis, acute cholangitis, abdominal wall abscess secondary to cholecystostomy tube

**Table 4:** Course of illness.

Course of illness	
Onset of shock	Day 8
Intubated	Day 8
Vasopressor initiation	Day 8
CVVH initiated	Day 11
First bone marrow biopsy	Day 11
Imaging suggestive of acalculus cholecystitis	Day 14
Percutaneous cholecystostomy	Day 15
Liver biopsy and second bone marrow biopsy	Day 17
Extubated	Day 18
Liver biopsy reveals acute cholangitis	Day 19
HLH diagnosis made	Day 22
Initiation of HLH dexamethasone therapy	Day 22
Initiation of HLH etoposide therapy	Day 24
Repeat vasopressor initiation	Day 30
CVVH discontinued	Day 44
Discharged to rehabilitation facility	Day 56
Treatment	2 doses of etoposide 40 mg/m^2^ and dexamethasone per HLH-94
Outcome	Complete remission (full recovery of cytopenias and return to baseline renal function)

**Table 5:** Antimicrobial course.

Antimicrobial	Day after admission
Azithromycin 500 mg oral one-time dose	Day 4
Azithromycin 250 mg oral daily	Day 5
Moxifloxacin 400 mg oral daily	Day 6
Vancomycin 1.25 g load and Piperacillin/Tazobactam 3.375 gm intravenous every 8 hours	Day 7-Day 11
Azithromycin 500 mg intravenous daily	Day 8-Day 11
Meropenem 1 g intravenous every 8 hours	Day 12-Day 24
Acyclovir 400 mg oral twice daily	Day 23-Day 34
Discontinuation of all antibiotics	Day 25
Vancomycin 2 g intravenous one-time dose	Day 30
Piperacillin/Tazobactam 3.375 g intravenous every 12 hours	Day 30-Day 36
Acyclovir 200 mg oral twice daily	Day 30-Day 47
Ampicillin 2 g intravenous daily	Day 37-Day 56
